# Optimized Parameters for Transducing the Locus Coeruleus Using Canine Adenovirus Type 2 (CAV2) Vector in Rats for Chemogenetic Modulation Research

**DOI:** 10.3389/fnins.2021.663337

**Published:** 2021-04-13

**Authors:** Latoya Stevens, Lars Emil Larsen, Wouter Van Lysebettens, Evelien Carrette, Paul Boon, Robrecht Raedt, Kristl Vonck

**Affiliations:** ^1^4BRAIN, Institute for Neuroscience, Department Head and Skin, Ghent University, Ghent, Belgium; ^2^Medical Imaging and Signal Processing, Department of Electronics and Information Systems, Ghent University, Ghent, Belgium

**Keywords:** locus coeruleus, chemogenetics, designer receptor exclusively activated by designer drugs, canine associated viral vector, neurological disorder

## Abstract

**Introduction:**

The locus coeruleus noradrenergic (LC-NA) system is studied for its role in various neurological and psychiatric disorders such as epilepsy and Major Depression Dissorder. Chemogenetics is a powerful technique for specific manipulation of the LC to investigate its functioning. Local injection of AAV2/7 viral vectors has limitations with regards to efficiency and specificity of the transduction, potentially due to low tropism of AAV2/7 for LC neurons. In this study we used a canine adenovirus type 2 (CAV2) vector with different volumes and viral particle numbers to achieve high and selective expression of hM3Dq, an excitatory Designer Receptor Exclusively Activated by Designer Drugs (DREADD), for chemogenetic modulation of LC neurons.

**Methods:**

Adult male Sprague-Dawley rats were injected in the LC with different absolute numbers of CAV2-PRSx8-hM3Dq-mCherry physical particles (0.1E9, 1E9, 5E9,10E9, or 20E9 pp) using different volumes (LowV = 3 nl × 300 nl, MediumV = 3 × 600 nl, HighV = 3 × 1200 nl). Two weeks post-injection, double-labeling immunohistochemistry for dopamine β hydroxylase (DBH) and mCherry was performed to determine hM3Dq expression and its specificity for LC neurons. The size of the transduced LC was compared to the contralateral LC to identify signs of toxicity.

**Results:**

Administration of Medium volume (3 × 600 nl) and 1E9 particles resulted in high expression levels with 87.3 ± 9.8% of LC neurons expressing hM3Dq, but low specificity with 36.2 ± 17.3% of hM3Dq expression in non-LC neurons. The most diluted conditions (Low volume_0.1E pp and Medium Volume_0.1E pp) presented similar high transduction of LC neurons (70.9 ± 12.7 and 77.2 ± 9.8%) with lower aspecificity (5.5 ± 3.5 and 4.0 ± 1.9%, respectively). Signs of toxicity were observed in all undiluted conditions as evidenced by a decreased size of the transduced LC.

**Conclusion:**

This study identified optimal conditions (Low and Medium Volume with 0.1E9 particles of CAV2-PRSx8-hM3Dq-mCherry) for safe and specific transduction of LC neurons with excitatory DREADDs to study the role of the LC-NA system in health and disease.

## Introduction

Through its widespread projections, the locus coeruleus (LC)–noradrenaline (NA) system modulates cortical, subcortical, cerebellar, brainstem, and spinal cord neuronal circuits influencing important physiological functions such as sleep, attention, memory, cognitive control and neuroplasticity (Samuels and Szabadi, 2008a,b; Szabadi, 2013). The LC-NA system is studied for its role in various neurological disorders such a depression, Parkinson’s disease, Alzheimer’s disease and epilepsy (Raedt et al., 2011; Grimonprez et al., 2015; Mather and Harley, 2016; Bari et al., 2020). Chemogenetics is a technique to specifically modulate brain structures and/or neuronal phenotypes using Designer Receptors Exclusively Activated by Designer Drugs (DREADDs) and greatly facilitates studying their contribution to behavior and disease. DREADDs are genetically modified G-protein coupled receptors inert for the endogenous ligand acetylcholine and activated by “designer drugs” such as Clozapine-N-Oxide (CNO), clozapine, deschloroclozapine (DCZ), compound 21 or JHU37152 and JHU37160 (Gomez et al., 2017; Bonaventura et al., 2019; Jendryka et al., 2019; Nagai et al., 2020).

A frequently used method to induce expression of DREADDs in LC neurons consists of injecting a viral vector, such as the adeno-associated virus (AAV) vector or canine adenovirus type 2 (CAV2) vector, in the LC region or its projection targets (Vazey and Aston-Jones, 2014; Hirschberg et al., 2017). Using PRSx8, a synthetic dopamine-β-hydroxylase (DBH) promotor, LC-specific DREADD expression is achieved (Hwang et al., 2001; Vazey and Aston-Jones, 2014). Apart from the type of viral vector and the promotor sequence, parameters such as injection site, volume and the amount of viral vector particles define cell-specific expression and specificity (Castle et al., 2016; Delbeke et al., 2017).

In this study, we compared different conditions for injecting CAV2-PRSx8-hM3Dq-mCherry viral vector in the LC to induce expression of the excitatory DREADD, hM3Dq, specifically in LC neurons. Our previous results demonstrated low cell-specific expression and specificity using the AAV2/7-PRSx8-hM3Dq-mCherry. We hypothesized that this is due to low tropism of the AAV2/7 viral particle for LC noradrenergic cells (Stevens et al., 2020). The CAV2 viral vector has been proven to efficiently transduce neurons by uptake both at the cell body and at the axonal terminals (Kremer et al., 2000; Soudais et al., 2000; Junyent and Kremer, 2015; Del Rio et al., 2019) and is validated to target the LC (Li et al., 2016; Hirschberg et al., 2017; Hayat et al., 2020). Pickering et al. demonstrated that CAV2 vectors containing the PRSx8 promotor can transduce LC neurons with high efficiency via direct or retrograde administration for opto-activation experiments (Li et al., 2016). However, Hayat et al. (2020) observed a substantial lower level of GtACR2 expression when compared to ChR2 using identical injection parameters. This indicates that transduction efficiency is amongst other factors dependent on the transgene itself. Additionally, to our knowledge no study describes the use of CAV2 carrying a plasmid to express the excitatory hM3Dq DREADD receptor. Since the transgene sequence itself can influence transduction efficiency, a reliable viral vector administration protocol is required when introducing a novel genetically modified receptor (Wang et al., 2019). When injecting a novel viral vector into a target area, safety of the administration has to be assessed, since viral vectors can induce toxicity with consequent cell loss (Sen et al., 2014).

By testing different injection volumes and numbers of CAV2-PRSx8-hM3Dq-mCherry viral vector particles to obtain optimal transduction efficiency with minimal aspecificity and toxicity in the LC of adult rats, we aim to achieve a condition where cell-specific modulation of the LC is feasible to study its role in regulating excitability of neural pathways.

## Materials and Methods

### Animals

Adult male Sprague-Dawley rats (Envigo, The Netherlands) were used in this study and treated according to European guidelines (directive 2010/63/EU). The local Ethical Committee on Animal Experiments of Ghent University (ECD 16/31) approved the study protocol. Animals were kept under environmentally controlled conditions: 12 h light/dark cycles with artificially dimmed light, temperature and relative humidity at 20–23°C and 40–60%, respectively, with food (Rats and Mice Maintenance, Carfil, Belgium) and water *ad libitum*. All animals were group-housed in Type IV cages (Tecniplast, Australia) on wood-based bedding (Carfil, Belgium). Cages were enriched with paper nesting material and cardboard tunnels (Carfil, Belgium).

### Viral Vector Administration

Animals (*n* = 43; 10–11 weeks old; 371 ± 72 g body weight) were anesthetized with a mixture of medical oxygen and isoflurane (5% for induction, 2% for maintenance, Isoflo, Zoetis, United Kingdom); body temperature was controlled using a heating pad. Rats were placed in a stereotaxic frame (Stoelting, United States) and the skull was exposed. Bregma was lowered 2 mm relative to lambda (15° head angle) to target the LC and avoid the transverse sinus. Using a Hamilton Neuros Syringe (Hamilton company, Nevada, United States) and Quintessential Stereotaxic Injection system (flowrate 150 nl/min, Stoelting, United States), three injections of CAV2-PRSx8-hM3Dq-mCherry vector solutions [5.9 × 10^9^ pp/μl undiluted, in phosphate-buffered saline (PBS)] were performed along the dorsoventral axis in the LC (3.9 AP, 1.15 ML relative to lambda, −5.8, −5.5, −5.3 DV from dura). The following injection conditions were tested: 3 × 300 nl containing 5E9 pp of CAV2 (LowV_5E9, *n* = 7, unilateral), 3 × 600 nl containing 10E9 pp CAV2 (MediumV_10E9, *n* = 8, unilateral), 3 × 1,200 nl containing 20E9 pp CAV2 (HighV_20E9, *n* = 5, unilateral), 3 × 300 nl containing 1E9 pp of CAV2 (LowV_1E9, *n* = 8, bilateral *n* = 5), 3 × 600 nl containing 1E9 pp CAV2 (MediumV_1E9, *n* = 6, unilateral), 3 × 300 nl containing 0.1E9 pp CAV2 (LowV_0.1E9, *n* = 4, bilateral) and 3 × 600 nl containing 0.1E9 pp of CAV2 (MediumV_0.1E9, *n* = 5, bilateral; Table 1).

**TABLE 1:** Overview of the different conditions: volume and number of physical viral particles.

**Group ID**	**# Animals**	**Volume**	**# Viral particles**	**Titer**
LowV_0.1E9	3	3 × 300 nl	0.1 × 10^9^	∼0.55 × 10^8^ pp/μl
MediumV_0.1E9	5	3 × 600 nl	0.1 × 10^9^	∼1.1 × 10^8^ pp/μl
LowV_1E9	7	3 × 300 nl	1 × 10^9^	∼0.55 × 10^9^ pp/μl
MediumV_1E9	5	3 × 600 nl	1 × 10^9^	∼1.1 × 10^9^ pp/μl
LowV_5E9	7	3 × 300 nl	5 × 10^9^	∼5.9 × 10^9^ pp/μl
MediumV_10E9	7	3 × 600 nl	10 × 10^9^	∼5.9 × 10^9^ pp/μl
HighV_20E9	5	3 × 1,200 nl	20 × 10^9^	∼5.9 × 10^9^ pp/μl

*The number of animals included in the analysis, without the four animals showing no clear hM3Dq expression (LowV_0.1E9 group, *n* = 1; LowV_1E9, *n* = 1; MediumV_10E9 group, *n* = 1; MediumV_1E9 group, *n* = 1).*

After each injection the syringe was left in place for an additional 5 min and then slowly retracted to avoid backflow. Two-component silicone gel (Kwik-Sil; World Precision Instruments, FL, United States) was applied to the craniotomy to protect the brain surface. After surgery, animals were subcutaneously (sc) injected with the non-steroidal anti-inflammatory drug meloxicam (1 mg/kg metacam, Boehringer Ingelheim, Germany) and lidocaine (5% Xylocaine gel, Astrazeneca, United Kingdom) was applied to the incision site to minimize discomfort. All animals recovered 2 weeks in their home cage to allow for optimal viral vector expression (Smith et al., 2016).

### Histology

#### Tissue Collection and Fixation

Animals were euthanized 2 weeks after viral vector injections with an overdose of sodium pentobarbital (200 mg/kg, i.p.) followed by a transcardial perfusion with PBS and subsequent paraformaldehyde (4%, pH 7.4). The brains were post-fixed in paraformaldehyde for 24 h and subsequently cryoprotected in a sucrose solution of 10% (1 day), 20% (±2 days), and 30% (±3 days) at 4°C until saturation was achieved, snap-frozen in isopentane and stored in liquid nitrogen at −196°C. After 1 h at −20°C, tissue was transferred to a Cryostat (Leica, Germany) and coronal cryosections of 40 μm were made.

#### Immunofluorescence Staining

Double label immunostaining for DBH and the mCherry tag fused to the hM3Dq receptor was performed to quantify specificity of hM3Dq expression, and evaluate possible damage to the LC. Twelve histological sections were selected per animal starting approximately 10.5 mm posterior to bregma, and keeping every third section (80 μm apart), toward anterior. Selected sections were rinsed twice for 5 min in distilled water (dH_2_O) followed by incubation in 0.5 and 1% H_2_O_2_ for 30 and 60 min, respectively, to block endogenous peroxidase activity. After washing twice for 5 min in PBS, sections were incubated for 45 min in blocking buffer (BB; 0.4% Fish Skin Gelatin (FSG) and 0.2% Triton X in PBS). For 1 h at room temperature and subsequently overnight at 4°C, sections were incubated in BB with primary antibodies to visualize noradrenergic LC neurons (mouse anti-DBH, 1:1000, clone 4F10.2, Merck) and mCherry tag (rabbit anti-red fluorescent protein, 1:1000, ROCK600-901-379, Rockland). The next day, sections were washed twice in BB for 10 min followed by 1 h incubation in BB with the secondary antibodies Alexa Fluor goat anti-mouse 488 nm (1:1000, Ab 150113, Abcam) and Alexa goat anti-rabbit 594 nm (1:1000, Ab 150176, Abcam) diluted in BB for 1 h. After washing twice in PBS for 5 min, a nuclear DAPI stain was performed. After two additional washing steps in PBS (2 × 5 min), sections were mounted on glass slides and cover slipped using Vectashield H1000 mounting medium (Vector Laboratories, United States) to prevent photobleaching.

#### Fluorescence Microscopy and Image Analysis

Glass slides were scanned using a Pannoramic 250 slide scanner equipped with a Plan Apochromat 40X objective and a pco.edge 4.2 4 MP camera to have overview images of all sections. Detailed images of the LC sections were acquired using the Olympus IX81 confocal microscope using Olympus Fluoview FV1000 software or a Nikon A1R confocal laser scanning microscope (Nikon Benelux, Brussels, Belgium) equipped with a Plan Apo VC 20X, 0.75 NA objective lens (Nikon). Images were exported as .nd2 and tiff files for post-processing.

For each rat, images of three tissue sections containing a clear LC nucleus were selected to qualitatively identify the presence and location of the hM3Dq expression. In bilaterally injected animals, both hemispheres were checked for expression and the first injected hemisphere was selected for further analysis, since this resembles the unilateral injected conditions. To assess possible signs of toxicity, in unilaterally injected animals, the injected LC was compared to the contralateral non-injected LC. Quantitative analysis in bilaterally injected animals was only performed in animals showing unilateral expression of the DREADD, to be able to compare the size of the DREADD-expressing versus the non-expressing LC. Bilaterally DREADD expressing animals were qualitatively assessed for signs of toxicity. Post-processing analysis was performed in Fiji ImageJ Software.

To determine cell-specific expression levels, hM3Dq-DBH colocalization was quantified by placing virtual markers on merged DBH^+^/DAPI images to identify the LC cells. These markers were then copied on the merged DBH^+^/mCherry images to determine the fraction of LC cells expressing hM3Dq-mCherry. To determine % aspecific hM3Dq expression, the total number of mCherry^+^ pixels were counted (# of white pixels after “selection of all pixels” and “invert” command on the 8-bit trict TIFF file), minus the ones present in the manually encircled LC and then divided by the total number of mCherry^+^ pixels.

To assess potential damage induced by the administration of the CAV2 viral vector and subsequent transduction, differences in DBH expression and LC size were compared between transduced and non-transduced hemispheres, since viral vector induced toxicity can induce cell loss. The DBH^+^ image (8-bit tiff file) was split into a transduced- and contralateral non-transduced site, followed by contrast enhancing using “autothreshold – default.” “Convert to mask” and “invert” commands to achieve a black background with white pixels. “Analyze particles tool” was used to select and encircle a cluster of white pixels and calculate the total area in pixels^2^ representing the size of both transduced and contralateral non-transduced LC. The size of both transduced and contralateral non-transduced LC was normalized to the average of the contralateral non-transduced LC within the animal (three sections) (%). Significant decreases in the size of the transduced LC compared to its contralateral non-transduced LC due to cell loss were interpreted as signs of toxicity (Thomas et al., 2001; Reimsnider et al., 2007; Sen et al., 2014).

### Statistical Analysis

Statistical analysis was performed using SPSS statistics 27 (IBM corporation, Armonk, New York, United States) and Graphs were made in Graphpad Prism 6. To compare LC-specific expression between conditions, the fraction of DBH^+^ cells expressing hM3Dq were fitted into a random effects linear mixed model as dependent variable with injection condition as fixed factor. The individual sections were selected as random intercept and variance components as covariance structure. Since the aspecific expression data is unbound and overdispersion is present, a generalized mixed model with negative binomial log link and with random intercept for animal ID was fitted to the data. The number of aspecific mCherry pixels was set as dependent variable with the log transformed all mCherry^+^ pixels as offset and the injection condition as fixed effect. To determine whether there was a significant difference in size of the transduced LC compared to the contralateral non-transduced LC per condition, a linear mixed model was performed with the normalized size as dependent variable, the condition and the side (transduced or non-transduced) and interaction as fixed factors and individual section as intercept. A significant decrease in transduced LC compared to its contralateral non-transduced LC was interpreted as toxicity. All data are represented as mean ± standard error of the mean. A *p*-value of < 0.05 was required for rejection of the null hypothesis.

## Results

### Viral Vector Expression

**FIGURE 1:**
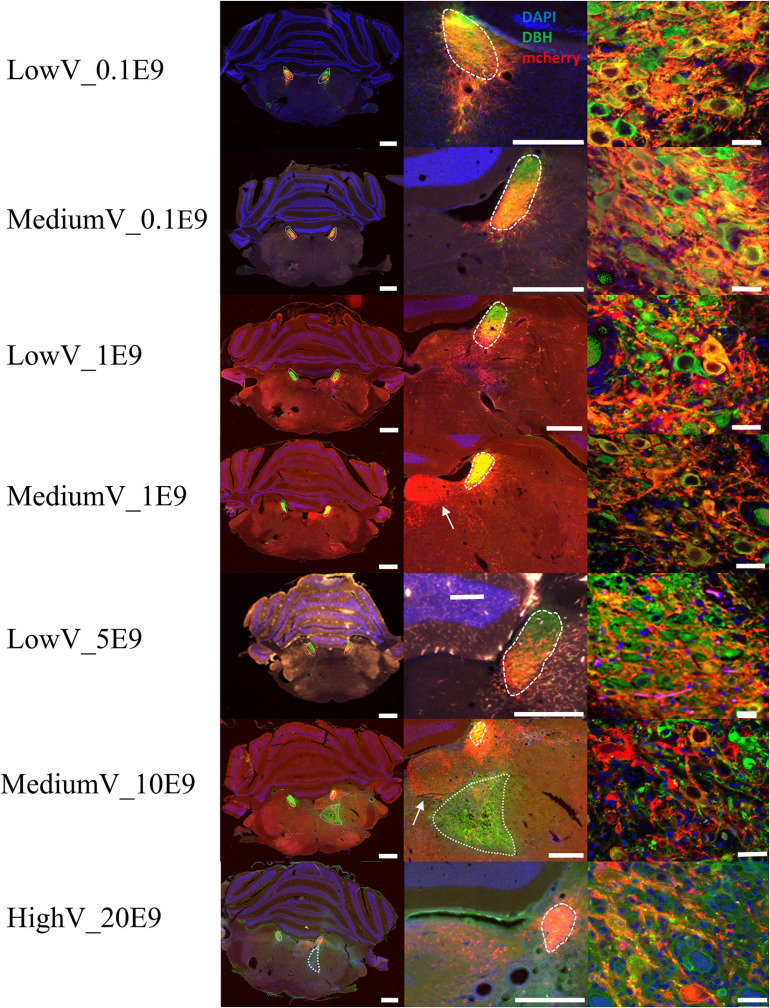
Representative visualization of hM3Dq expression pattern in LC after injection of CAV2-PRSx8-hM3Dq-mCherry in LC for each condition. Each row represents a condition. The first column represents an overview of an LC containing tissue section; the second column shows a more detailed view of hM3Dq expression in the region of LC. The last column shows a confocal image of DBH^+^/hM3Dq^+^ cells. LC is encircled with a dashed line. Aspecific hM3Dq expression is indicated by a white arrow. Ectopic DBH expression is indicated by the dotted line. LC-NA neurons and projections are visualized using primary anti-DBH antibody (green, AF 488 nm) and expression of hM3Dq DREADD is visualized using anti-RFP (red, AF 594 nm), cell nuclei are stained with DAPI (blue). Scale bar is represented at each image, left column 1,000 μm, middle column 500 μm and right column 25 μm.

In all but four animals (*n* = 1 of LowV_0.1E9 group, *n* = 1 of LowV_1E9, *n* = 1 of MediumV_10E9 group, *n* = 1 of MediumV_1E9 group), clear hM3Dq expression was present in the soma and axonal projections of LC neurons (Figure 1). In only 6/13 (46%) of the bilaterally injected animals, bilateral expression of hM3Dq expression was observed, possibly due to off-target administration (Supplementary Figure 1, injection tract visible; Rorabaugh et al., 2017).

**FIGURE 2:**
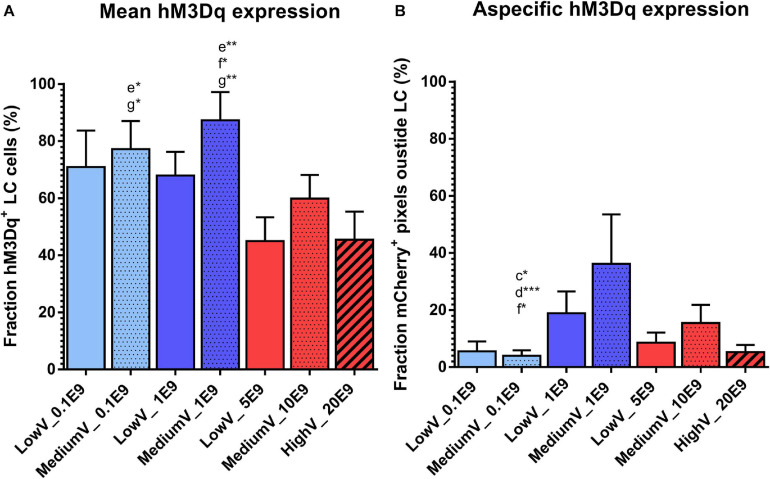
Transduction efficiency of CAV2-PRSx8-hM3Dq-mCherry viral vector to express hM3Dq in LC. **(A)** Mean hM3Dq expression in DBH positive cells after manual cell count. MediumV_1E9 induces the highest level of hM3Dq expression compared to LowV_5E9, MediumV_10E9, and HighV_20E9. MediumV_0.1E9 condition shows similar levels, significantly higher compared to LowV_5E9 and HighV_20E9. **(B)** Mean aspecific hM3Dq expression. In the MediumV_0.1E9 condition the lowest level of aspecific expression is observed compared to LowV_1E9, MediumV_1E9 and MediumV_10E9. GLM negative binomial statistical analysis showed that the chance of an mCherry + pixels detected outside LC is, respectively, 4.8, 9.1, and 3.9 times higher in the LowV_1E9, MediumV_1E9 and MediumV_10E9 condition compared to MediumV_20E9. Bars represent the mean ± SEM per condition. Pattern inside the bars represent the injected volume: low, 3 × 300 nl = no pattern; medium, 3 × 600 nl = dotted pattern; high, 3 × 1200 nl = striped pattern. Colors of the bars represent the dilution: undiluted (5,10,20E9 pp) = red; diluted to 1E9 pp = dark blue; diluted to 0.1E9 pp = light blue. Letters above the bar represent significant difference with the corresponding condition (a: the first bar from the left, in alphabetical order with the last bar being g). Stars added to the letters indicate the level of significance (*p < 0.05; **p < 0.01, ***p < 0.001).

The fraction of LC neurons expressing hM3Dq was highest in the MediumV_1E9 condition (87.3 ± 9.8%) and significantly different from the LowV_5E9 (44.9 ± 8.3%, *p* = 0.002), MediumV_10E9 (59.8 ± 8.3%, *p* = 0.041), and HighV_20E9 group (45.5 ± 9.8%, *p* = 0.005). MediumV_0.1E9 group (77.2 ± 5.9%) has significant higher expression compared to the LowV_5E9 (44.9 ± 8.3%, *p* = 0.018) and HighV_20E9 group (45.5 ± 9.8%, *p* = 0.029; Figure 2A).

A generalized mixed negative binomial model estimates the rate of aspecific hM3Dq expression in the LowV_1E9 (18.9 ± 7.6%) and MediumV_1E9 condition (36.2 ± 17.3%) to be 4.8 times (95%CI 1.4–16.4, *p* = 0.014) and 9.1 times (95%CI 2.4–34.7, *p* = 0.001) higher compared to the MediumV_0.1E9 condition (4.0 ± 1.9%), showing the lowest levels of aspecificity. The aspecific expression in the MediumV_10E9 condition (15.5 ± 6.3%) is estimated to be 3.9 times higher (95% CI 1.1 −13.5, *p* = 0.031) compared to the MediumV_0.1E9 condition (4.0 ± 1.9%) (Figure 2B and Supplementary Table 1 Fixed coefficients).

### Signs of Toxicity

**FIGURE 3:**
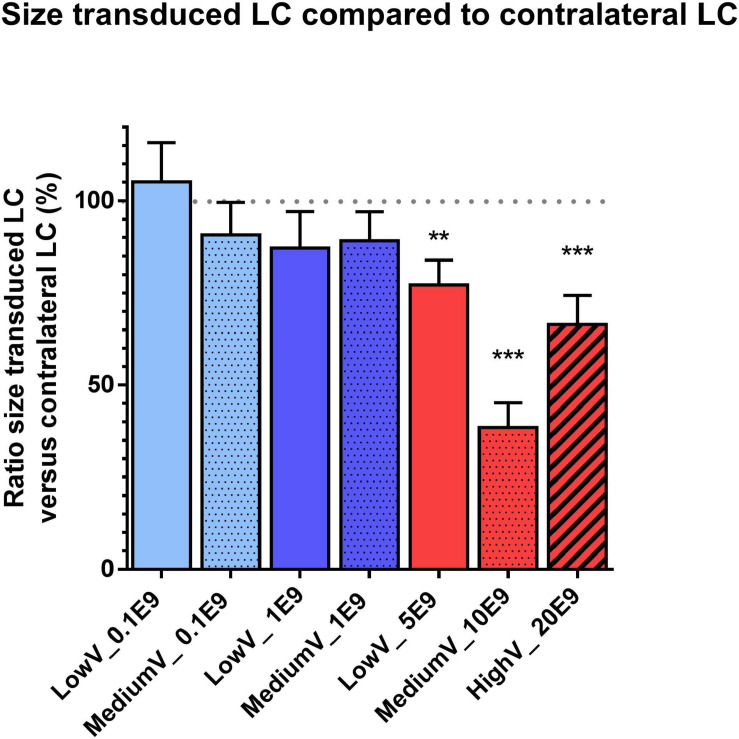
Graphical representation of the size of the transduced LC compared to the contralateral non-transduced LC. A significant decrease in size was observed in all undiluted conditions with the highest decrease in the MediumV_10E9 condition (transduced LC was 38.5 ± 6.7% of the contralateral LC size, *p* < 0.001). A significant decrease in size of transduced LC was also observed in the LowV_5E9 (77.2 ± 6.7, *p* = 0.002) and HighV_20E9 (66.4 ± 7.9, *p* < 0.001) condition. Bars represent the mean difference in LC size ± SEM per condition normalized to the contralateral non-transduced LC (represented by dotted line). Pattern inside the bars represent the injected volume: low, 3 × 300 nl = no pattern; medium, 3 × 600 nl = dotted pattern; high, 3 × 1,200 nl = striped pattern. Colors of the bars represent the dilution: undiluted (5,10,20E9 pp) = red; diluted to 1E9 pp = dark blue; diluted to 0.1E9 pp = light blue. (**p < 0.01, ***p < 0.001).

**FIGURE 4:**
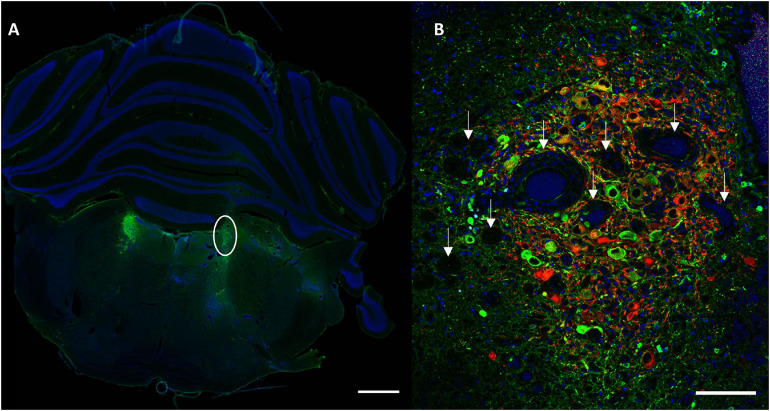
Representative section visualizing signs of toxicity. **(A)** A clear difference in size of the transduced LC (right, encircled) compared to contralateral in animal injected with MediumV_10E9 condition. Only cell nuclei (DAPI, blue) and DBH^+^ neurons (primary antibody anti-DBH, AF 488, green) are visualized in this image. **(B)** A clear decrease in number of LC neurons, in combination with the presence of lesions and vacuoles (indicated by the white arrows). LC-NA neurons and projections are visualized using primary anti-DBH antibody (green, AF 488 nm) and expression of hM3Dq DREADD is visualized using anti-RFP (red, AF 594 nm), cell nuclei are stained with DAPI (blue). Scale bar left image 1,000 μm and right image 100 μm.

Conditions with the highest number of viral particles were associated with a clear difference in DBH expression and size of the transduced LC compared to the contralateral LC. Quantitative comparison of transduced versus contralateral non-transduced LC size showed a significant size reduction in these conditions. Toxicity was significantly high in the MediumV_10E9 condition (*p* < 0.001), showing a significant decrease in the size of the transduced LC compared to its contralateral LC. The size of injected LC was 38.5 ± 6.7% of the contralateral LC volume. A significant reduction in size of transduced LC was also observed in the LowV_5E9 (77.2 ± 6.7%; *p* = 0.002) and HighV_20E9 (66.4 ± 7.9%; *p* < 0.001) conditions compared to the contralateral non-transduced LC (Figure 3). Qualitative analysis of tissue sections from the MediumV_10E9 and HighV_20E9 condition showed additionally to decreased LC size, ectopic DBH expression in the transduced brainstem and clear lesions in the injected LC (Figures 1A,B MediumV_10E9, HighV_20E9; Figure 4). Qualitative analysis of the bilaterally hM3Dq expressing animals did not show clear visible signs of toxicity in the LowV_0.1E9, LowV_1E9 and MediumV_0.1E9 condition as confirmed by quantitative analysis of the unilateral expressing animals of these groups (Representative image Supplementary Figure 2).

## Discussion

This study evaluated different conditions for injection of CAV2 viral vector to obtain expression of hM3Dq DREADD in a high number of LC neurons with limited aspecific expression and toxicity.

All conditions resulted in hM3Dq expression in LC noradrenergic neurons but differences in expression specificity and toxicity were observed. Transduction levels are the highest when diluted conditions, 0.1E9 pp and 1E9 pp of CAV2-PRSx8-hM3Dq-mCherry at the target site, are used with average expression ranging from 67.9 ± 8.3 to 87.3 ± 9.8%. This is much higher compared to our previous AAV2/7 approach (20.6 ± 2.3%; 5.99 × 10^9^ GC/μl) (Stevens et al., 2020) and similar to other groups using the CAV2 viral vector to induce expression of Channelrhodopsin under control of a PRSx8 promotor at conditions similar to ours (3 injections of 400nl, a total of 10^9^ viral particles at the target site) observing expression levels of 83 ± 3.4% (Hayat et al., 2020). Low expression levels of hM3Dq were observed with the highest numbers of viral particles (>1E9 pp). A similar decrease in expression levels associated with increasing titers have been described upon pontospinal injections of AAV for retrograde labeling of noradrenergic neurons (Howorth et al., 2009) and after injections of an adenovirus vector in striatum (Thomas et al., 2001). Injection of high viral vector titers can result in blood-brain barrier changes and leakage of viral particles in systemic circulation as observed after AAV injection in the substantia nigra of mice, resulting in lower expression levels (Van der Perren et al., 2011). Injecting more than 1E9 CAV2 particles also resulted in signs of toxicity with decreased size of LC, ectopic DBH expression and the presence of lesions. Excess load of viral particles can induce cellular stress and disrupt the homeostasis of the endoplasmatic reticulum (ER). Once ER stress is induced, and proteins do not properly fold, the unfolded protein response (UPR) pathways are activated to restore the cellular machinery. This includes activation of immune pathways with recruitment of macrophages, astrocytes and microglia and infiltration of antigen non-specific T cells. These immune response can result in clearance of viral particles as well as substantial loss of astrocytes and neurons (Thomas et al., 2001; Reimsnider et al., 2007). Additionally, when a point of no return is reached, UPR will induce apoptosis and autophagy leading to cell loss (Sen et al., 2014). Next to high presentation of viral proteins and transgene, high levels of the PRSx8 promotor might also induce changes in LC-NA physiology due to sequestration of Phox2 proteins at the multiple Phox2 binding sites in the promotor sequence. These proteins are necessary for maintaining normal NA phenotype, possibly explaining changes in DBH expression and even cell loss (Hwang et al., 2005). Although the ectopic DBH expression was only observed in animals injected with high titer conditions, we cannot rule out that this phenomenon is possibly due to non-specific binding of the antibody to compromised cells (Buchwalow et al., 2011).

The CAV2 viral vector, a non- human derived viral vector, is described to be safer in use for long-term experiments and eventual translation to the clinic, due to the absence of a pre-existing immune response (Kremer et al., 2000; Del Rio et al., 2019), however, our observations indicate that the use of high titers of CAV2-PRSx8-hM3Dq-mCherry can induce signs of toxicity with tissue damage and concurrent lower transduction efficiency potentially due to a combination of innate immune responses, cellular stress and phox2 protein sequestration (Thomas et al., 2001; Hwang et al., 2005; Sen et al., 2014).

Although injecting 1E9 CAV2 particles (MediumV_1E9 condition) did not result in LC damage, a significant presence of aspecific hM3Dq expression (36.2 ± 17.3%) was observed. By further reducing the number of injected CAV2 particles to 0.1E9, efficient transduction of LC neurons was preserved (>70%) while aspecific expression was significantly reduced to levels below 5% in the MediumV_0.1E9 and LowV_0.1E9 condition. Previous studies using the CAV2 viral vector to induce PRSx8-driven expression of ChR2, PSAM and GtACR2 reported comparably low levels of aspecific expression (0–2%) (Hickey et al., 2014; Hirschberg et al., 2017; Xiang et al., 2019; Hayat et al., 2020). Viral vectors (both AAV and CAV2) containing PRSx8 driven plasmids are widely used and validated to induce selective transduction in noradrenergic neurons both in mice and rats (Vazey and Aston-Jones, 2014; Fortress et al., 2015; Li et al., 2016; Hirschberg et al., 2017; Kane et al., 2017; Rorabaugh et al., 2017; Vazey et al., 2018; Cope et al., 2019; Xiang et al., 2019; Hayat et al., 2020). In a previous study, using AAV2/7 viral vector to transduce cells with the PRSx8-hMD3q-mCherry construct, we observed low levels of hM3Dq expression in LC (20.6 ± 2.3%) with high levels of aspecific expression (26.0 ± 4.1%) outside the LC (Stevens et al., 2020). These observations indicate that CAV2 is a more efficient tool than AAV2/7 to introduce genes in LC neurons and that PRSx8 can result in important leakage of expression in non-catecholaminergic neurons. The latter effect is thought to be (partially) mediated by Phox2 regulatory elements in the promotor sequence activated by Phox2 transcription factors also present in non-catecholaminergic neurons (Hwang et al., 2001; Bruinstroop et al., 2012). In our study, off-target hM3Dq expression was assessed using pixel values instead of manually counting mCherry^+^/DBH^–^ cells. To quantify aspecific expression levels in a similar fashion as the cell-specific hM3Dq levels quantification, confocal images of the entire section should be acquired to detect any possible off-target expression and provide the ability for manual cell counting. As aspecific expression was not the primary hypothesis of this study resulting in the lack of confocal image data outside of the LC region, quantification of the DBH^–^/mCherry^+^ pixels of the epifluorescence whole section images served as a valid proxy for off-target expression.

In a subset of the injected animals no expression was observed (four in total) and in a subset of bilaterally injected animals unilateral expression was observed, possibly due to off-target administration, similar to other studies describing the difficulties of targeting a small brainstem nucleus and observing unilateral expression after bilateral LC administration of the viral vector (Kane et al., 2017; Rorabaugh et al., 2017). Animals with bilateral hM3Dq expression could not be included for quantitative analysis of toxicity. In these animals of the LowV_0.1E9, LowV_1E9, and MediumV_0.1E9 conditions qualitative analysis did not show major signs of toxicity as observed with the high titer conditions.

Our results indicate that introducing a new genetic product into a target region using viral vectors should be approached with caution and thorough titration with control for toxicity. A few papers describe these phenomena after injection of adenoviral or adeno-associated viral vectors, however, not much is reported about CAV2 (Thomas et al., 2001; Reimsnider et al., 2007; Sen et al., 2014). Since the use of CAV2 viral vectors in future clinical trials is considered because of its known advantages such as high tropism for neurons, ability for retrograde transport and lack of existing immune response in patients (Kremer et al., 2000; Junyent and Kremer, 2015; Del Rio et al., 2019), further research including its safety at higher titers is necessary.

We conclude that direct injection of 0.1E9 CAV2-PRSx8-hM3Dq-mCherry viral vector particles in LC of adult rats is a suitable approach for near complete transduction of LC neurons while keeping aspecific expression and toxicity low. This will allow future chemogenetic modulating of LC to unravel its role in regulating the excitability brain networks and in specific neurological diseases.

## Data Availability Statement

The raw data supporting the conclusions of this article will be made available by the authors, without undue reservation.

## Ethics Statement

The animal study was reviewed and approved by The Ethical Committee on Animal Experiments of Ghent University.

## Author Contributions

LS, WVL, RR, and KV contributed to the study design and analyzed plan. LS performed the experimental work and obtained histological data. LS and WVL performed data and statistical analysis. LS, RR, and KV prepared the manuscript. All authors reviewed the manuscript.

## Conflict of Interest

The authors declare that the research was conducted in the absence of any commercial or financial relationships that could be construed as a potential conflict of interest.
