# Supplementary material for: Optimized Parameters for Transducing the Locus Coeruleus Using Canine Adenovirus Type 2 (CAV2) Vector in Rats for Chemogenetic Modulation Research
Source: Front Neurosci. 2021 Apr 13;15:663337. doi: 10.3389/fnins.2021.663337 (PMC8076532; doi:10.3389/fnins.2021.663337)
Supplement: Supplementary file 1 [file Table_1.DOCX]

Supplementary Material

# Supplementary Figures and Tables


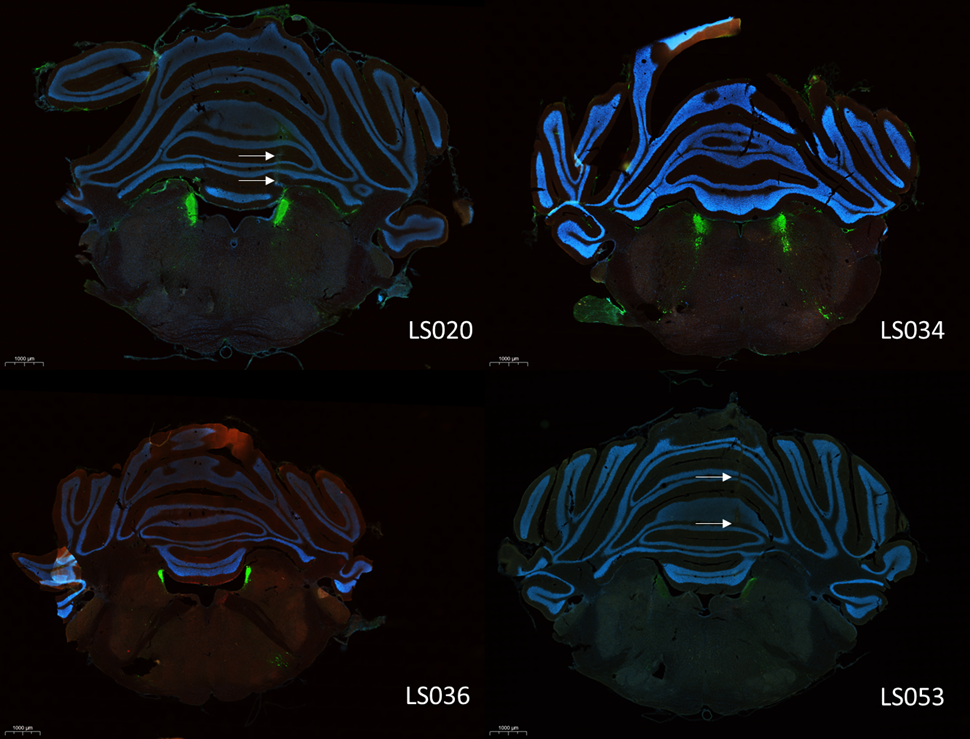


Supplementary Figure 1 Overview of animals without clear hM3Dq expression. LS020 (MediumV_10E9), LSO34(LowV_1E9), LSO36(MediumV_1E9) and LSO53 (LowV_0.1E9) show no hM3Dq expression probably due to off target injection. The injection tract (indicated by white arrow) is visible in LSO20 and LSO53 and indicates the injection to be medial from LC, possibly ending in the fourth ventricle explaining absence of hM3Dq expression.


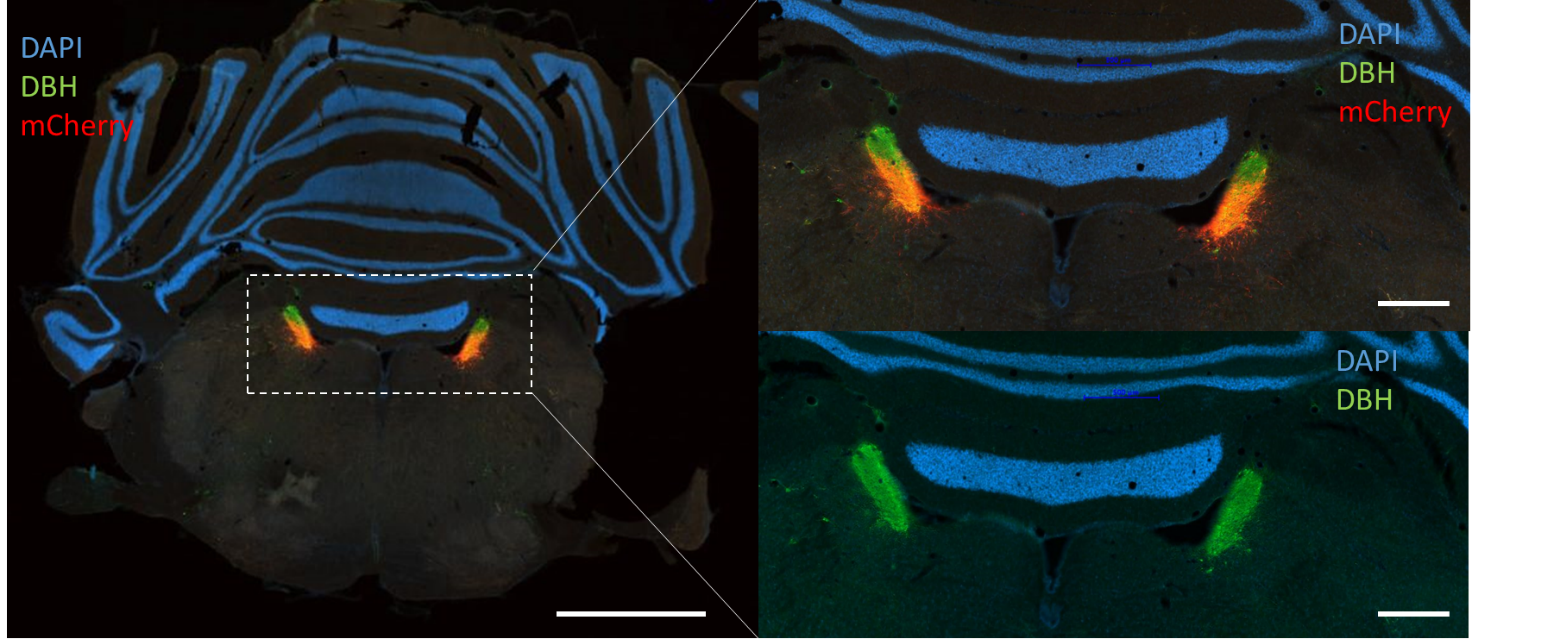


**Supplementary Figure 2 Representative image of bilaterally injected animal** bilaterally expressing hM3Dq DREADD. Left panel. Overview of section (LowV_0.1E9). Right upper panel gives an overview of bilateral hM3Dq expression. Right lower panel showing LC without hM3Dq, indicating no clear signs of toxicity. Qualitative analysis reveals identical size, no cell loss or lesions. Scale bar left 2000µm, right 500µm.

**Supplementary Table 1. Fixed coefficients of the generalized mixed model with negative binomial log link analysis of the aspecific expression dataset.**
